# Erdafitinib-induced hand-foot syndrome

**DOI:** 10.1016/j.jdcr.2026.07.074

**Published:** 2026-09-09

**Authors:** Kelly Centeno, Julia Fortier, Pooja Gurnani, Kiran Motaparthi

**Affiliations:** a Donald and Barbara Zucker School of Medicine at Hofstra/Northwell, Hempstead, New York; b Department of Dermatology, University of Florida, Gainesville, Florida

**Keywords:** chilblain lupus erythematosus, drug reaction, erdafitinib, FGFR inhibitor, hand-foot syndrome, mucositis, onycholysis

*To the Editor:* We read with interest the manuscript “Chilblain lupus erythematosus associated with erdafitinib therapy in a patient with metastatic bladder cancer” by Truong et al^1^ and wish to offer an alternative interpretation of these clinicopathologic findings: hand-foot syndrome (HFS) induced by fibroblast growth factor receptor (FGFR) inhibitor.

The patient described by Truong et al was a 57-year-old man who developed painful necrosis of the distal digits, onycholysis, erythema, and ulceration of the volar fingertips after 3 months of therapy with erdafitinib, an FGFR inhibitor, for metastatic urothelial carcinoma. Histopathology showed interface dermatitis, which can be present in chilblain lupus erythematosus (CLE), but may also reflect the cytotoxic effect of erdafitinib. Additionally, there was a notable absence of papillary edema, a typical feature of CLE. Low-power images were not published, but CLE also typically demonstrates a dense perivascular and perieccrine lymphocytic infiltrate. While not exclusive of CLE, the patient’s rheumatologic workup was negative, including antinuclear antibodies and lack of typical Raynaud phenomenon. Furthermore, onycholysis was prominent in this patient but is not a characteristic feature of chilblains.

Instead, the range of findings aligns closely with the cutaneous toxicity profile of FGFR inhibitors, specifically HFS. As detailed by Lacouture et al in a comprehensive review of dermatologic adverse events associated with selective FGFR inhibitors, HFS in this context is localized to the digits.^2^ While HFS due to cytarabine and anthracyclines demonstrates central involvement of the palms and soles, HFS due to FGFR inhibitors typically affects the volar aspect of the distal digits and lateral aspects of the hands and feet, accompanied by onycholysis and secondary paronychia. Mucositis is also reported following treatment with FGFR inhibitors, observed in up to 58% of erdafitinib-treated patients with urothelial carcinoma.^1^

We present an additional case of erdafitinib-associated HFS with similar clinical features:

A 73-year-old man with a history of metastatic mucinous cystadenocarcinoma developed mucositis and a painful eruption on the hands and feet after 14 weeks of erdafitinib therapy. Physical examination revealed erosions of the labial mucosa, prominent onycholysis, erythema of the distal digits, and painful paronychia (Fig 1, Fig 2, Fig 3, Fig 4). After discussion with the patient’s oncologist, erdafitinib was continued at a reduced dosage (4 mg daily). The patient was treated with topical metronidazole cream, 0.75% twice daily, and cephalexin 500 mg daily for 30 days (doxycycline was avoided due to dysphagia secondary to his cancer). Vinegar soaks to the fingernails and toenails were also recommended.

**Fig 1:**
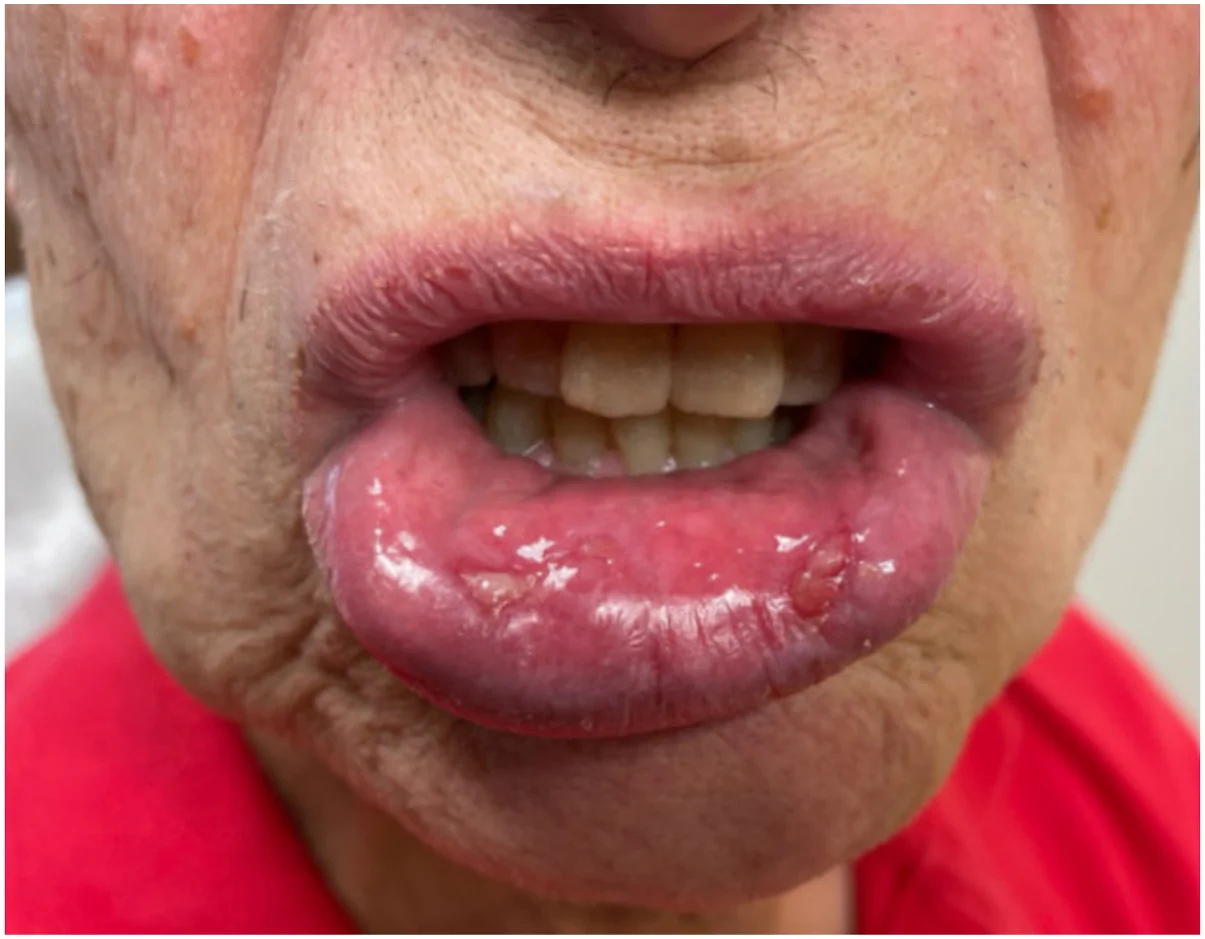
Erosions of the labial mucosa in a patient with erdafitinib-associated hand-foot syndrome.

**Fig 2:**
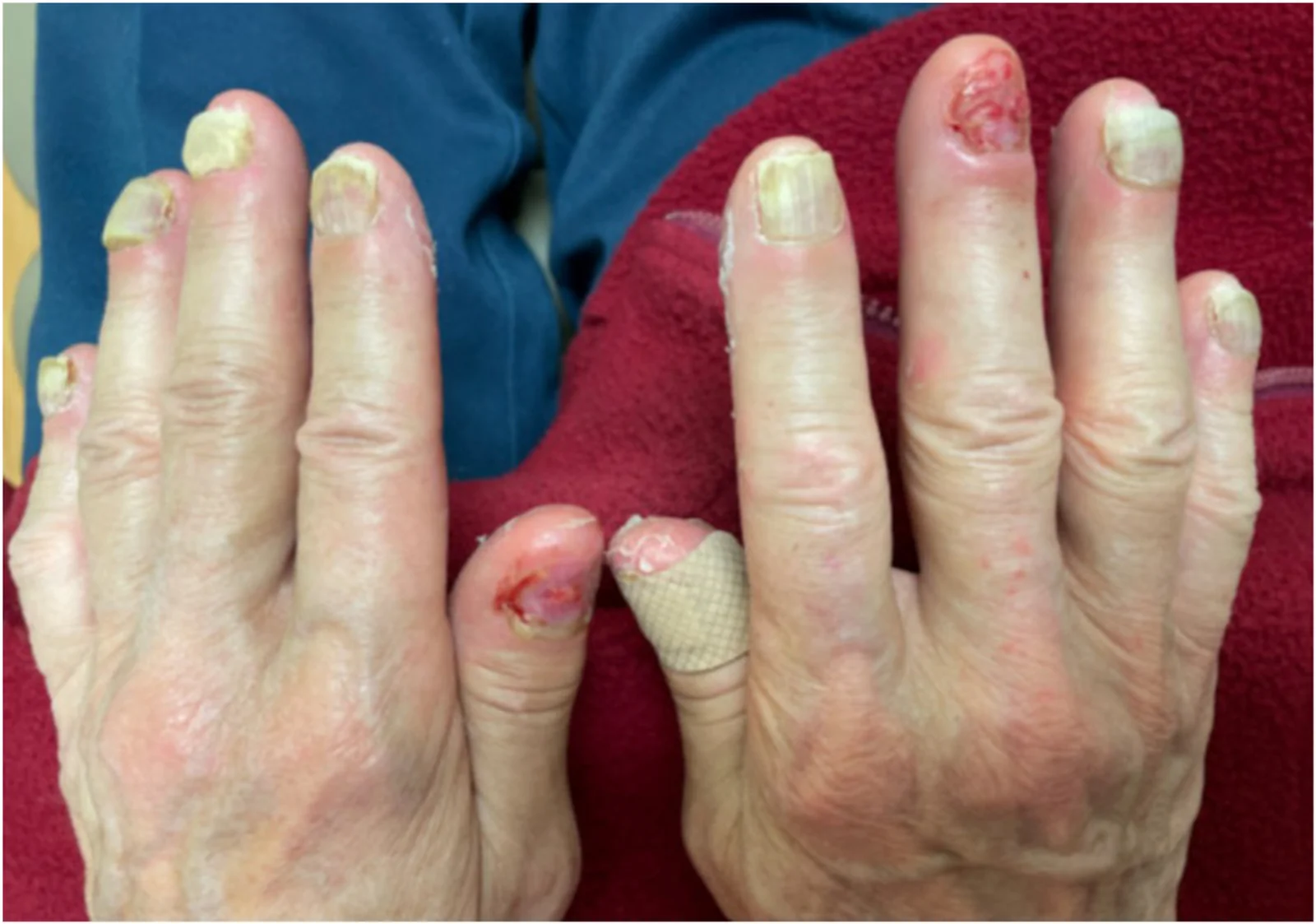
Prominent onycholysis and paronychia of the fingernails with erythema of the distal digits in a patient with erdafitinib-associated hand-foot syndrome.

**Fig 3:**
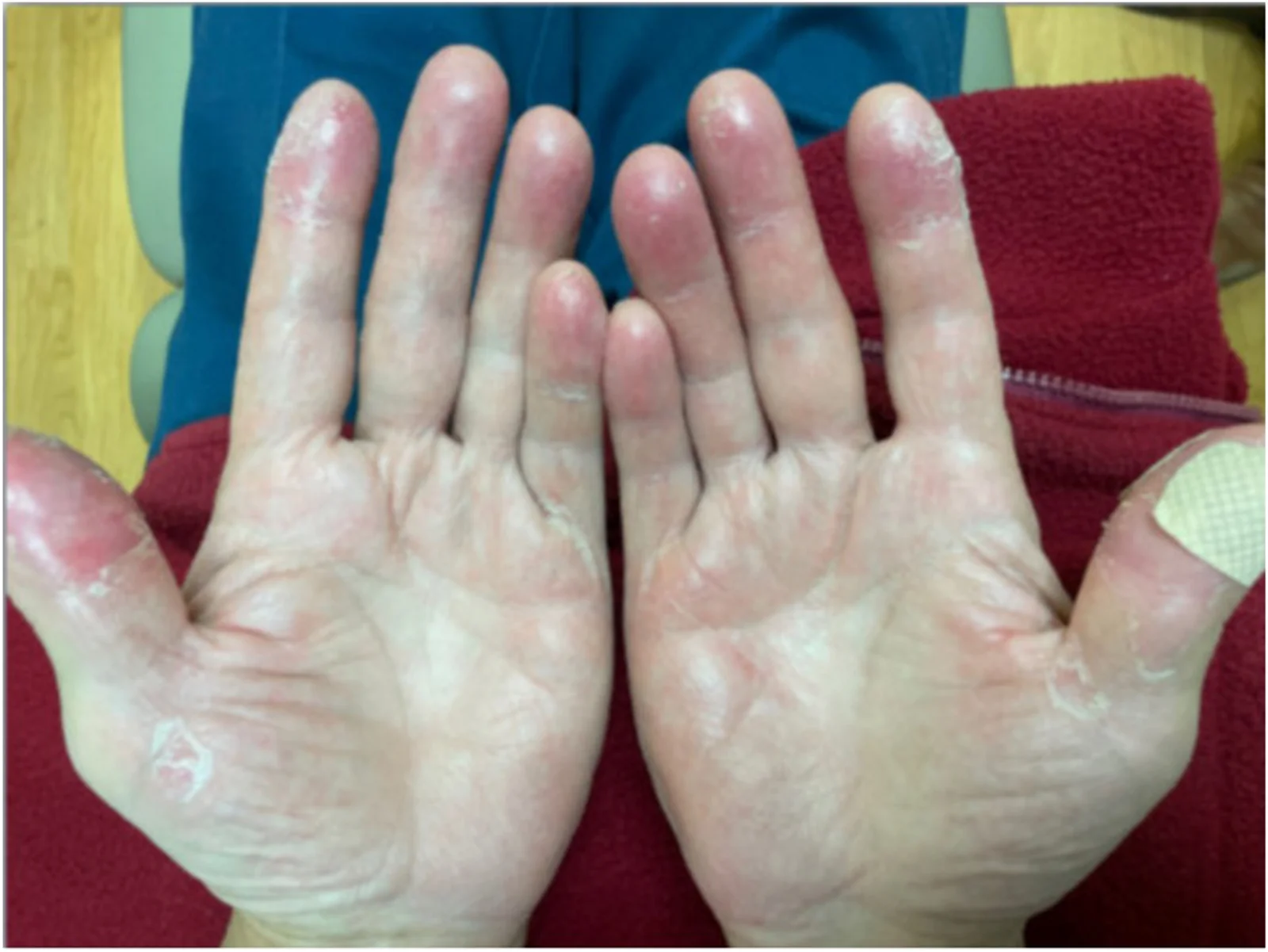
Erythema with focal hyperkeratotic plaques on the volar fingertips and lateral palms in a patient with fibroblast growth factor inhibitor-induced hand-foot syndrome.

**Fig 4:**
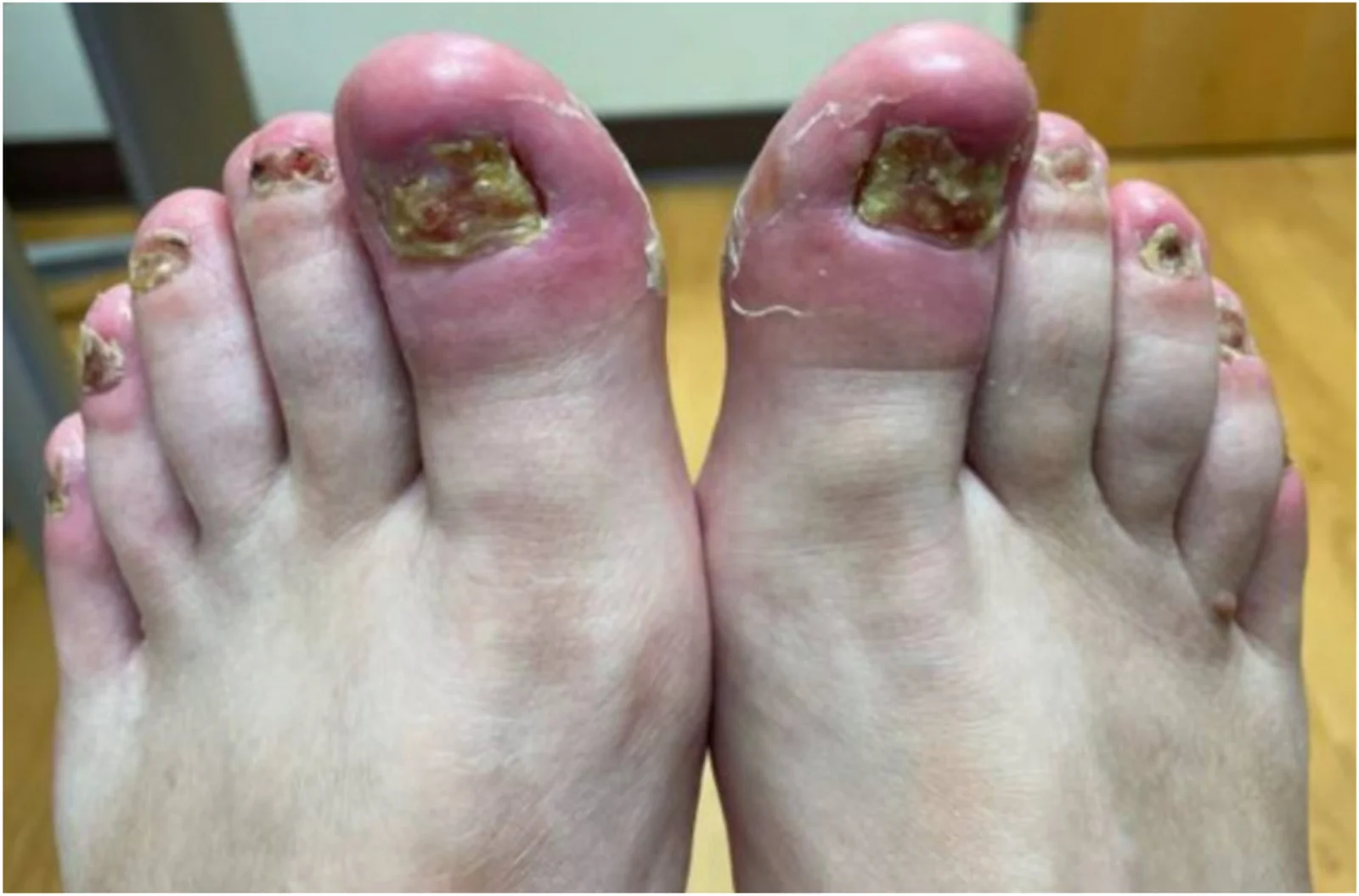
Paronychia and onycholysis in a patient with fibroblast growth factor inhibitor-induced hand-foot syndrome.

The patient described by Truong et al and our patient both show cutaneous adverse effects of FGFR inhibitors: erythema and ulceration of the distal fingers, onycholysis, and paronychia. Mucositis, when present, also aids in diagnosis of FGFR toxicity. Increased awareness of FGFR inhibitor-induced HFS may aid in recognition as well as cessation or decrease in dosage of the FGFR inhibitor when appropriate.

## Conflicts of interest

None disclosed.
